# Central nervous system involvement in sarcoidosis

**DOI:** 10.1590/0100-3984.2014.0113

**Published:** 2015

**Authors:** Vinicius Silles Machado, Nivaldo Adolfo Silva Junior, Luciano Souza Queiroz, Fabiano Reis, Danilo dos Santos Silva, Flavia Fagundes Bueno, Ana Carolina Coan

**Affiliations:** 1 Universidade Estadual de Campinas (Unicamp), Campinas, SP, Brazil.

**Figure 1:**
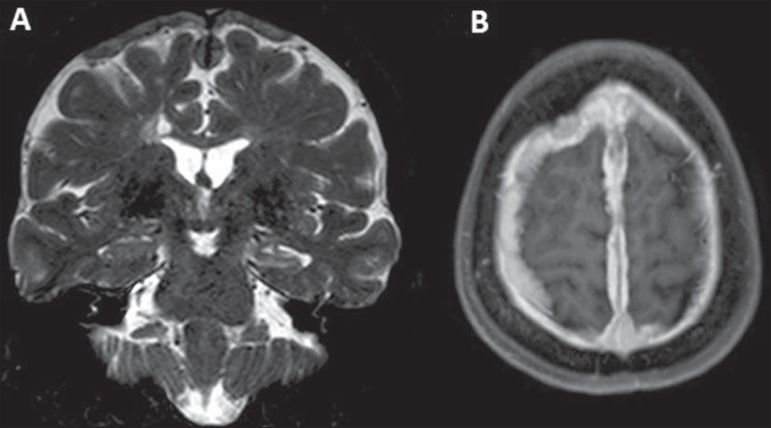
**A:** Coronal magnetic resonance imaging – T2-weighted sequence demonstrating diffuse pachymeningeal thickening most prominent at the high convexity and extending bilaterally toward the falx, with predominance of hyposignal in association with reduction in volume and hypersignal of the left hippocampus (mesial sclerosis). **B:** Paramagnetic contrast-enhanced axial T1-weighted sequence showing diffuse and homogeneous pachymeningeal enhancement.

*Dear Editor*,

A 51-year-old female patient complained of mild frontotemporal headache of insidious-onset for two years. One year ago, she had an episode of focal, tonic-clonic seizures (with right lower limb paresthesia) and was prescribed carbamazepine. Cerebrospinal fluid demonstrated increased protein levels and intrathecal immunoglobulin (IgG) synthesis, suggesting an inflammatory component. Magnetic resonance imaging was performed (Figure 1).

Sarcoidosis is a multisystem disease of unknown etiology characterized by noncaseating granulomatous inflammation^(1)^. There is a genetic predisposition, with T-lymphocyte receptor activation by some unknown antigen. The disease affects preferentially the respiratory system^(1)^. In the lungs, granulomas are observed in the interstitial compartment, showing a perilymphatic distribution along the peribronchovascular sheaths, interlobular septa and pleural surface^(1)^.

It is estimated that in about 5% to 15% of cases sarcoidosis affects the central nervous system. Rarely the patient presents with exclusively neurological manifestations like in the present case. Most commonly, neurosarcoidosis is observed in cases of disseminated disease^(2)^.

The clinical manifestations of neurosarcoidosis are pleomorphic. Cranial nerve compromise, visual alterations, headache, weakness, paresis, paresthesia, psychiatric alterations and signs of meningeal irritation may be observed. Although rare, symptoms of diabetes insipidus such as polydipsia and polyuria may also occur due to the involvement of the hypothalamus and hypophysis. In cases of spinal cord involvement, weakness of lower limbs and other nonspecific signs of myelopathy are observed^(3)^.

Although sarcoidosis may manifest in all the regions of the central nervous system, it is most commonly seen in the skull base, hypothalamus, pituitary and optic chiasm^(4)^. At magnetic resonance imaging, a common finding is intraparenchymal lesions with hypersignal on T2-weighted and FLAIR sequences, generally multifocal, periventricular, subcortical or in the deep white matter. Such findings can hardly be differentiated from vasculitis or demyelinating diseases. Intraparenchymal lesions are generally located near the areas with leptomeningeal involvement (with enhancement by paramagnetic contrast medium), and may be either single or multiple, possibly also involving cranial nerves^(4)^.

**Figure 2:**
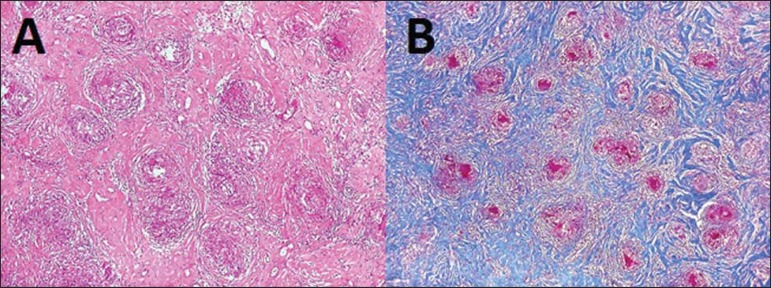
**A:** Biopsy of thickened area of the dura mater showing numerous noncaseating epithelioid and giant cell granulomas and predominantly lymphocytic inflammatory infiltrate intermingled with dense collagenous fibrosis. **B:** Masson’s trichrome stain: granulomas (pink) against dense collagenous connective tissue (blue).

Like in the present case, diffuse pachymeningeal thickening may be observed, with hyposignal on T2-weighted, isosignal on T1-weighted sequences and contrast enhancement. Thus, differential diagnoses such as neurotuberculosis, dural lymphoma, meningioma en plaque, IgG4 deposition disease, pseudotumor, adenocarcinoma metastasis, Wegener's granulomatosis, idiopathic hypertrophic pachymeningitis might be considered, requiring biopsy to define the etiology. Simultaneous dural and leptomeningeal involvement is rarely observed^(4)^. In the present case, the anatomopathological findings corresponded to typical noncaseating granulomas in the pachymeninges (Figure 2). Intracranial hypotension is another differential diagnosis to be considered, generally presenting with diffuse pachymeningeal thickening, but with hypersignal on T2-weighted sequences (in the present case, hyposignal was observed on T2-weighted sequences).

A consensus is still to be reached on the treatment for sarcoidosis. In cases where the patient is symptomatic the treatment is initiated with high doses of corticosteroids, gradually reduced along the treatment up to complete withdrawal^(3)^.
